# The Radiation-Induced Regenerative Response of Adult Tissue-Specific Stem Cells: Models and Signaling Pathways

**DOI:** 10.3390/cancers13040855

**Published:** 2021-02-18

**Authors:** Paola Serrano Martinez, Lorena Giuranno, Marc Vooijs, Robert P. Coppes

**Affiliations:** 1 Department of Biomedical Sciences of Cells and Systems-Section Molecular Cell Biology, University of Groningen, University Medical Center Groningen, 9713 AV Groningen, The Netherlands; p.serrano.martinez@umcg.nl; 2 Department of Radiation Oncology, University of Groningen, University Medical Center Groningen, 9700 RB Groningen, The Netherlands; 3 Department of Radiation Oncology (Maastro), GROW School for Oncology and Developmental Biology, Maastricht University Medical Centre, P.O. Box 616, 6200 MD Maastricht, The Netherlands; l.giuranno@maastrichtuniversity.nl

**Keywords:** radiotherapy, stem cells, signaling pathways, regeneration

## Abstract

**Simple Summary:**

A common side effect of radiotherapy is the impairment of integrity and functionality of the co-irradiated surrounding normal tissue. Homeostasis and regeneration of many organs are maintained by specific stem/progenitor cells. Radiation can harm these resident stem/progenitor populations involving the disruption of the signaling cascade of pathways known to normally sustain stem/progenitor cellular activity. This review describes the currently existing models used to study the response of stem/progenitor cells to irradiation and the key signaling pathways involved during solid tissue-specific stem/progenitor driven regeneration.

**Abstract:**

Radiotherapy is involved in the treatment of many cancers, but damage induced to the surrounding normal tissue is often inevitable. Evidence suggests that the maintenance of homeostasis and regeneration of the normal tissue is driven by specific adult tissue stem/progenitor cells. These tasks involve the input from several signaling pathways. Irradiation also targets these stem/progenitor cells, triggering a cellular response aimed at achieving tissue regeneration. Here we discuss the currently used in vitro and in vivo models and the involved specific tissue stem/progenitor cell signaling pathways to study the response to irradiation. The combination of the use of complex in vitro models that offer high in vivo resemblance and lineage tracing models, which address organ complexity constitute potential tools for the study of the stem/progenitor cellular response post-irradiation. The Notch, Wnt, Hippo, Hedgehog, and autophagy signaling pathways have been found as crucial for driving stem/progenitor radiation-induced tissue regeneration. We review how these signaling pathways drive the response of solid tissue-specific stem/progenitor cells to radiotherapy and the used models to address this.

## 1. Introduction 

One of the main limitations of radiotherapy (RT) is the damage induced to the healthy tissue positioned unavoidably in the radiation field. Radiation-induced side effects can be linked to the loss of tissue stem cells (SCs) and damage accumulation in the remaining stem/progenitor cells. This may result in acute or late adverse effects depending on the number of surviving stem/progenitor cells. A better understanding of SC response and the pathways that orchestrate the regenerative response of the stem/progenitor pool in tissues to RT can help to predict unavoidable toxicity and aid to prevent or repair radiation-induced damage. In this review, we summarize our current understanding of the pathways that may promote solid tissue SC response to RT and the current models used to characterize RT response.

## 2. Models to Study SC Response to Radiation 

**Figure 1:**
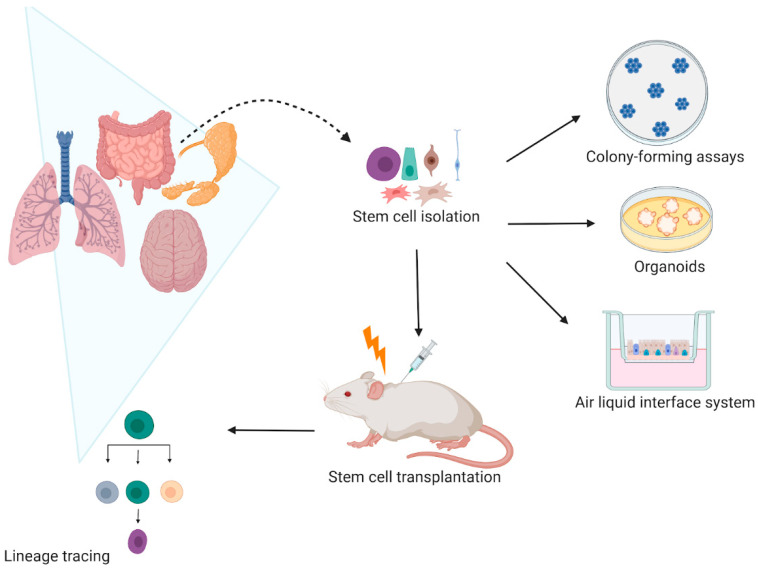
Current models use to assess stem cell radiation response in vivo and in vitro. In vitro the self-renewal potential of stem cells is evaluated by assessing their colony-forming efficiency in clonogenic assays. The stem cell self-renewal potential is also studied in three-dimensional (3D) organoids and air–liquid interface (ALI) systems that not only allow stem cell radiation response studies, but also their differentiation capacity upon irradiation. In vivo, the stem cell lineage tracing remains the most used model that enables to specifically mark stem cells and follow their cell fate. Therefore, it is possible to characterize how irradiation affects the stem cell self-renewal and differentiation capacity. Created with BioRender.com.

Many studies have assessed the self-renewal and differentiation potential of SCs upon irradiation (IR). These include two-dimensional (2D) and three-dimensional (3D) in vitro clonogenic studies of cell lines, spheroids, and organoids, replating assays, and in vivo lineage tracing (Figure 1). Although many IR studies have used submerged culture procedures, such as clonogenic and replating studies, they are unable to mimic the actual in vivo microenvironment and organ functionality [1,2]. The 3D models, such as spheroids, organoids, air–liquid interface (ALI) systems, and organ-on-chips recapitulate the organ structure, seem to better reflect the patient-specific response compared to in vitro 2D cell line models and enable assessment of in vitro SC responses to IR [3].

### 2.1. Organoids

Organoids are derived from highly self-renewing tissue SCs that can differentiate in all of the lineages and retain the genetic and phenotypic characteristics of both tumor and normal tissue in vitro. Passaging of the organoids enriches for cell population with self-renewing capacities, such as stem and progenitor cells. SC radiation response may be reflected by the next passage organoid forming potential, which measures the SC self-renewal potential. Fluorescence-activated cell sorting of cell surface SC markers (e.g., P63+, Lgr5+, Ngfr+, Nkx2+) allows the identification, isolation, and enrichment of tissue-specific stem/progenitor cells that can be cultured to study the mechanisms involved in SC DNA repair and self-renewal after radiation in experimental conditions closer to the in vivo situation [4,5].

Furthermore, patient-specific tissue-derived organoids are not hampered by interspecies differences, which is one of the limitations of animal models and can be genetically modified to study a specific pathway involved in radiation response. The combination of organoids with gene expression modulation and genome editing techniques supports the ease of organoid studies and therefore their versatility as a model system [6]. An example of results that would never have been discovered in a 2D model is described in the study of Gao et al. [7]. They showed how the use of 3D-cultured mammospheres revealed important differences in radiation-induced senescence between cancer and non-tumorigenic epithelial cells. Moreover, 20 Gy irradiation leads to high enrichment of CD44+/CD24−/low subpopulation of putative mammary epithelial stem cells while the same dose in MCF-7 mammary cancer cells did not increase the fraction of this subpopulation. These results suggest that phenotypic plasticity appears to be highly regulated in non-tumorigenic cells and SC enrichment occurs at high doses (20 Gy). Therefore, the deregulation of stem cell enrichment may play a role in carcinogenesis, providing an advantage to cells that are more capable of being reprogrammed to a stem-like state [7].

### 2.2. ALI System

The air–liquid interface system (ALI system) is an example of how better models allow more in-depth studies [8]. ALI is a transwell system that allows differentiation of the upper airway basal lung SCs into a polarized mucociliary pseudostratified epithelium in vitro and enables the study of the basal and luminal cell response to radiation [9]. Indeed, this system better resembles the in vivo structure of the lung and allows the coculture with different cell types such as tumor cells, fibroblasts, or immune cells. For instance, lung SC response to radiation was studied by Giuranno et al. using this model [8,10]. Inhibition of the Notch SC pathway was shown to increase lung SC self-renewal upon IR, contributing to a more intact epithelium as assessed in a completely differentiated setting [8]. This effect was not observed in 2D models, which do not permit the culture of the differentiated cells, suggesting the contribution of the luminal cells to SC response to RT [10]. This indicates that the choice of the right model may be crucial for translating preclinical research findings to clinical practice. However, there is still an urgent need to better characterize these in vitro models and develop new ones to replace animal models and the unsatisfactory 2D systems widely used in radiation studies. However, these models lack the blood vessel, immune, and innervation systems that have been involved in the SC response [11].

### 2.3. Co-Culture Models

Co-culture models have, for example, been used to assess the colony-forming ability of progenitor cells isolated from mice exposed to isotropic doses of high and low linear energy transfer (LET) IR [12]. Fluorescent lung epithelial cells were isolated at different time points post-IR and plated in a 3D co-culture system containing unirradiated non-fluorescent fibroblasts. Whole-body exposure to isotropic doses of ionizing radiation resulted in a loss of progenitor cell colony-forming ability, 1-day post-exposure. The progenitor loss was more pronounced after high let ionizing radiation suggesting that exposure to high-, but not low-LET radiation leads to prolonged defects in the ability of progenitors to proliferate and repair the airway epithelium.

Co-culture of irradiated human dermal microvascular endothelial cells and human adipose-derived SCs showed reduced levels of interleukin (IL) 6, fibroblasts growth factor, intercellular adhesion molecule-1, and vascular cellular adhesion molecule-1 supporting the clinical observation that adipose-derived SCs have a stabilizing effect when injected into irradiated wounds [13]. Other studies have shown that fibroblasts co-cultured with pericytes and mesenchymal SCs increased vessel densities in both irradiated and non-irradiated groups, underlining how the choice of a co-culture system provides answers to more complex questions concerning the interaction between different cell types [14]. Neurons have been shown to self-organize around salivary epithelial cells in co-culture models, in a similar fashion to what occurs in native tissue [15]. Therefore, the nervous system coculture model should be developed as a potential platform for studying neuron-salivary epithelial cell interactions for predicting radiation responses. 

These data indicate that current models are an improvement and may link 2D experiments with in vivo experiments, but that further development is necessary to assess the complete scale of normal tissue effects after IR.

### 2.4. Lineage Tracing Models

Among the models widely used to characterize radiation response, in vivo SC lineage tracing has been extensively adopted as it enables to specifically mark SCs and follow their cell fate. The CreERT2/LoxP system is widely used in lineage tracing studies. In this system, a tissue- or cell-specific promoter drives the expression of Cre recombinase fused with the ligand-binding domain of the estrogen receptor (ER), which can excise a stop sequence flanked by LoxP sites, leading to the expression of the cell-specific reporter gene, when induced by an estrogen receptor antagonist such as tamoxifen. Inducible Cre-ER systems offer the advantage of the spatiotemporal control of the pathway to be studied. Thus, it will be possible to turn a specific receptor on and off specifically in the SCs and follow their cell fate. Cre-ER systems have been used to study Wnt and Notch signaling pathways in the intestine, such as in Lgr5 positive cells, where lineage specification, repair, and the regenerative response upon IR were shown to be Notch-dependent [16]. The role of Notch and Wnt signaling was also investigated using a knockin allele engineered at the mouse Lyz1 (Lysozyme) locus, to perform detailed Paneth cell-lineage tracing. After IR, Paneth cells acquire a SC phenotype by activation of the Notch but not Wnt/β-catenin pathway, suggesting that Notch activation is sufficient to induce a fate change [17]. Temporal analysis of irradiated Paneth cells detected phospho-Stat3 levels, which can be activated by c-Gas-Sting pathway, IL10, and IL12 [18,19]. Indeed, single-cell sequencing showed that the Lgr5-expressing population in the crypt contained radioresistant intestinal SCs (ISCs) required for epithelial regeneration after IR and that Yes-associated protein 1 (Yap1)/Wnt signal balanced surviving crypt epithelial cells and determines the cellular contribution to epithelial regeneration [20].

The lineage tracing system was also extensively studied in glands such as prostate and salivary glands (SGs). Using a Nestin-Cre driver, it was shown that Nestin+ Ng2+ prostate cells identify self-renewing SCs, which are radioresistant and contribute to organ regeneration after 25 Gy IR [21]. In SGs, in vivo lineage tracing of Keratin 5 (Krt5) CreER and Axin2 CreERT2 showed that after 15 Gy IR, regenerated acinar cells are derived from both differentiated acinar and duct cells demonstrating that cellular plasticity contributes to regeneration in injured SGs [22].

The lineage tracing inducible system offers the advantages that the Cre-mediated recombination can be controlled in time, through tamoxifen administration. This is advantageous because only the desired cell population at a specific time point will be labeled and traced. Therefore, in vitro systems, such as organoids, organ-on-chip, ALI systems have been developed to overcome some of the animal study limitations such as the large number of animals needed, the costs, ethical issues, and relevance to human studies.

### 2.5. SC Therapy

SC therapy has been widely used in preclinical models not only to unravel the mechanism behind SC radiation response, but also to temper the radiation effects after exposure, as it offers the opportunity of replacing lost cells and promotes tissue repair [23]. SC therapy may be a therapeutic opportunity for the treatment of radiation-induced toxicity. However, only a few preclinical studies are available for radiation-induced adverse effects, which clearly show a time-dependent effect depending on the temporal sequence in which they are administered after RT. Bone marrow-derived mesenchymal SCs (MSCs) and adipose tissue-derived MSC have been isolated, transplanted in preclinical models, and characterized. MSCs successfully migrate towards the injury site, and through anti-inflammatory and anti-apoptotic properties can boost the remaining SC proliferation and subsequently repopulate the damaged organ [24,25]. Salivary gland stem cell (SGSC) (xeno-)transplantation in recipient mice, with locally irradiated SGs, allows engraftment of SCs and restores SG architecture and saliva secretion [26]. 

Stem cell transplantation has also been used to ameliorate cognitive dysfunction in the rat brain undergoing RT. In an athymic rat model subjected to cranial IR, intrahippocampal transplantation of human neural SCs provides long-lasting cognitive benefits by restoring the expression of plasticity-related ARC (activity-regulated cytoskeleton-associated) proteins. Therefore, human neural SCs transplantation promotes the long-term recovery of host hippocampal neurons reducing cognitive dysfunctions after IR [27].

Due to the complexity of the underlying interactions between biological tissue and ionizing radiation, more advanced modeling techniques will likely become necessary for SC RT response. Patient-derived SC models, reproducing clinical conditions as closely as possible, are needed to provide information that can be translated from bench to clinics. Improving animal models, transplantation methods, engraftment, and imaging may facilitate the use of SCs in the clinic to prevent long-term radiation-induced loss of tissue function. 

## 3. Signaling Pathways That Contribute to SC Radiation Response 

### 3.1. Notch Signaling Pathway 

**Figure 2:**
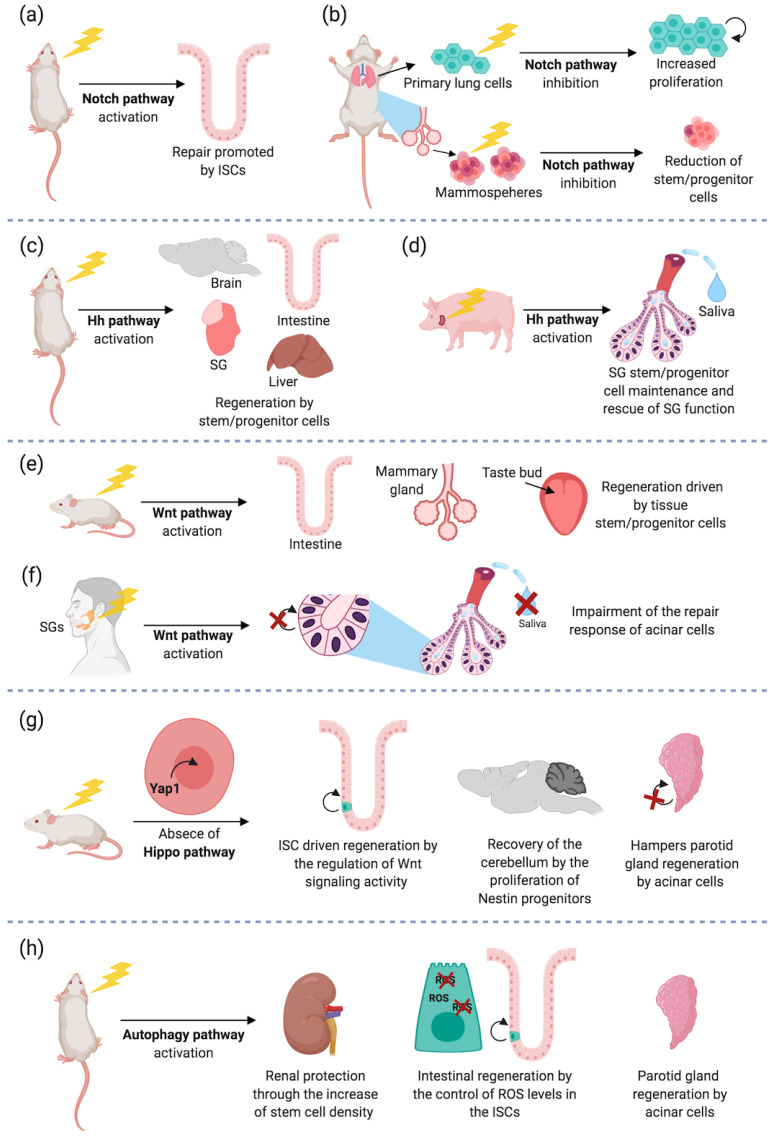
Principal signaling pathways involved in the response of stem/progenitor cells to irradiation. (**a**) The activation of the Notch signaling pathway in intestinal stem cells (ISCs) drives the post-irradiation in vivo regeneration of the murine gut. (**b**) Notch inhibition hampers the maintenance of stem/progenitor cells of the murine mammary gland after irradiation in vitro, while it promotes the response of mouse lung stem/progenitor cells. (**c**) The activity from the hedgehog (Hh) signaling pathway orchestrates the regeneration driven by stem/progenitor cells in the mouse brain, intestine, salivary gland (SG), and liver. (**d**) SG radiation-induced dysfunction is rescued by cues from the Hh signaling pathway in a pig model. (**e**) Activation of the Wnt canonical pathway results in stem/progenitor driven regeneration of the murine intestine, taste bud, and mammary gland post-irradiation. (**f**) The activation of the Wnt pathway hampers the repair response of human acinar cells to irradiation. (**g**) The absence of Hippo signaling through Yap1 nuclear translocation promotes the intestinal and cerebellar recovery after irradiation. However, nuclear Yap1 localization impairs the parotid gland regeneration. (**h**) The activation of the autophagy pathway protects the mouse kidney, intestine, and parotid gland from irradiation. Reactive oxygen species (ROS). Created with BioRender.com.

The Notch pathway has been extensively studied in the SC field although only a few papers have investigated the effects of the Notch pathway in the radiation response of normal tissues. The Notch pathway is a highly conserved signaling cascade that controls morphogenesis and homeostasis in adult tissues through receptor-ligand interactions on adjacent cells [28,29]. The Notch pathway encodes for four Notch receptors that, upon ligand binding, undergo sequential proteolytic cleavages, which culminate with the release of the Notch intracellular domain, which translocates into the nucleus and activates gene transcription [30]. In the intestine, Notch plays a leading role in regulating epithelial cell fate resulting in enterocyte differentiation [31,32,33], while its inhibition promotes secretory cell differentiation, including goblet, Paneth, and endocrine cells [34,35,36,37]. It has been shown that Notch signaling is necessary for ISC proliferation, self-renewal, and repair [34,38]. Notch pathway inhibition resulted in reduced expression of the ISC marker Olfm4, decreased numbers of Lgr5+ SCs, and reduced SC proliferation [39,40]. Carulli et al. [41], found that Notch signaling is crucial for the maintenance of the Lgr5+ crypt base columnar SC population upon IR. Moreover, 12 Gy whole-body IR in Notch1 and Notch2 conditional knockout (cKO) mice showed reduced SC proliferation, weight loss, and abnormal secretory distribution. An altered epithelial architecture was observed, suggesting that while neither receptors are critical for proliferation during normal homeostasis, they are both required for post-injury proliferation [41]. Similar results were obtained in another in vivo study where, Klf5 a downstream target of Notch, was shown to be required for the Lgr5 SC regenerative response after IR injury. Regenerating crypts were markedly reduced in Lgr5-klf5 mice after 12 Gy IR showing that Klf5 is required for crypt cells to dedifferentiate and regenerate the intestinal epithelium following radiation injury [16]. The importance of Notch for intestinal regeneration after IR (Figure 2a) was also observed by Qu et al. [42], who showed that administration of the Notch/γ-secretase inhibitor DAPT, 24 h before 12 Gy total body IR significantly reduces the number of the regenerative crypts with associated loss of Dclk1+ ISCs. Recent studies have suggested that activated Notch signaling stimulates Paneth cell plasticity during injury-induced regeneration [17,43]. Lineage tracing of Paneth cells showed that after IR Notch is required to acquire SC features and to boost their proliferation and regeneration [17]. Therefore, therapeutic inhibition of Notch to radiosensitize tumor cells should be approached with caution due to the important role it plays in homeostasis and repair.

The role of the Notch pathway was investigated in the mammary SCs grown as mammosphere although the studies show controversial results. Dontu et al. [44] reported that Notch signaling activation with DSL peptide resulted in a 10-fold increase of mammosphere-forming efficiency. On the other hand, Bouras et al. [45] showed that Notch inhibition by knockdown Cbf1 in CD29hi/CD24+ cells resulted in increased transplantation efficiency, suggesting that the Notch pathway restricts the pool of mammary stem/progenitor cells. Tao et al. [46] showed that Notch inhibition through the γ-secretase inhibitor DAPT in 5 Gy irradiated mammospheres drastically reduced the number of mammary stem progenitors (Figure 2b). This study suggests that Notch promotes the expansion of irradiated mammary SCs. The discrepancy between these studies may be related to the models used for the studies (wild type versus genetically modified cells, pharmacological inhibition versus knockdown) and the population of SCs specifically targeted.

The role of the Notch pathway in the lung SC in response to RT has been investigated in only a few papers [8,10]. Giuranno et al. [8] showed that Notch inhibition increased the proliferation of the irradiated primary lung SCs, reduced DNA damage, and contributed to a more intact epithelium (Figure 2b). 

However, more studies are needed to unravel the mechanism of action behind the protective effect of inhibiting Notch in the lung undergoing radiation damage.

### 3.2. Hedgehog Pathway 

The Hedgehog (Hh) pathway is one of several cross-talking intercellular signaling pathways, which plays an important role in SC regeneration after RT (Figure 2c,d) and has a crucial role in SG, liver, and brain SC survival. Hh signaling is activated by the derepression of Smoothened (Smo) protein. Smo is a G-coupled transmembrane protein, resulting from the interaction between Hh ligands and the receptor Patched (Ptch), and is mediated by Gli transcription factors [47]. Although the role of Hh in the adult SG is marginal, it is activated during functional regeneration after duct ligation and promotes epithelial proliferation after damage. Loss of SGSCs, due to the high sensitivity of the SGs to RT, causes xerostomia, characterized by RT irreversible hyposalivation and reduced quality of life [48]. 

After RT, transient Hh activation significantly rescued SG function, promoting salivary stem progenitor cell maintenance, parasympathetic innervation, and expression of related genes, including those in Bmi1 and Chrm1/HB-EGF pathways [49,50]. Similarly, Hu et al., showed that transient activation of Hh pathway in the SG, by inducible expression of Hh transgene in Krt5+ epithelial cells or adenovirus-mediated intragland transfer of Hh gene, rescued IR-induced hyposalivation [51]. Therefore, transient activation of the Hh represents a promising strategy to promote SG regeneration after RT and prevent xerostomia (Figure 2c). The Hh pathway regulates SC regeneration by interacting in a coordinated manner with other pathways such as Fgf and Wnt signaling. Hh signaling regulates FGF8 protein expression which rescues SGs from Hh inhibition in ex vivo models [52]. Wnt activation upregulates Hh expression underlining the interesting cross-talk between the two pathways. The role of the Hh pathway was also investigated in a pig model of RT-induced hyposalivation (single dose 20 Gy on the parotid area) where transient activation of Hh signaling, by intragland transfer of Hh gene, preserved the function of the SGs (Figure 2d) [53]. Furthermore, it mitigated the microvascular damage, promoted angiogenesis during tissue repair, and regeneration and induced the secretion of pro-angiogenic factors, preserving VEGFA expression. Furthermore, intraglandular Shh gene delivery alleviated RT-induced cellular senescence by reducing p21/Cdkn1A and increasing Ki67 proliferating cells, and improving parasympathetic innervation [51]. Thus, Hh gene transfer is a feasible approach to mitigate the detrimental effect of RT on SG function.

Furthermore, the Hh signaling pathway is activated in the damaged liver and regulates tissue reconstruction (Figure 2c) [54]. In mice receiving 20 Gy whole-body IR, increased Hh signaling promoted proliferation of progenitor cells and activated transformation of hepatic stellate cells into myofibroblasts, which then contribute to hepatic fibrogenesis in RT-injured livers [54]. The Hh pathway was also upregulated in the late liver response to 6 Gy and its inhibition blocked the proliferation of progenitor cells and reduced fibrogenic gene expression, suggesting that the pathway is associated with both acute and late response of the liver to IR [54,55]. Similar results were shown by using fractionated RT in mice irradiated with 6 Gy for a total of 30 Gy. Hh was upregulated in the irradiated mice and promoted fibrogenic stimuli in the injured liver with an expansion of the responsive progenitors [56]. In the intestine, administration of 1-[(4-nitrophenyl)sulfonyl]-4-phenylpiperazine (compound 5), which activates Hh signaling by binding to the transmembrane domain of Smoothened, 24 h after IR for 5 consecutive days promoted crypt regeneration 96 h after injection with a significant increase in the number of proliferating (Ki67-positive) cells within the crypt compartment of the small intestine. Furthermore, compound 5 increased ISC proliferation in vitro in early enteroid passages, which still contain stromal components and is quite likely to create an anti-inflammatory environment permissive for ISC expansion or interconversion [57]. The same compound 5, when given after cranial IR, preserves the neural stem/progenitor cell population (Figure 2c), inhibits microglial activation, mitigates radiation-induced neuroinflammation as shown by reduced IL6 secretion, and prevents radiation-induced cognitive impairment in mice without compromising radiation antitumor effect, suggesting that this compound could be used to mitigate radiation side effects in brain tumor patients undergoing RT [58].

Given the significant role of Hh signaling in SC regeneration and repair after injury and its crucial role in cancer progression, more in-depth research is needed to develop more effective interventions that inhibit the Hh pathway in cancer while preserving its function in healthy tissue. 

### 3.3. Wnt Canonical Signaling Pathway 

The Wnt signaling pathway drives the development and homeostatic SC maintenance of multiple tissues, such as the mammary gland [59], intestine [60], and taste bud [61]. Activation is initiated after the binding of Wnt ligands to Frizzled receptors and Lrp5/6 co-receptors, which will trigger the release of β-catenin from the Axin-APC-GSK-3b destruction complex. This will allow the stabilization and translocation of β-catenin to the nucleus where it will bind to Lef/Tcf transcription factors, to activate transcription of target genes like Axin2, c-Myc, Cyclin D1, Lgr5 [61,62,63]. 

After IR, the tight modulation of the Wnt signaling has been shown as crucial for the promotion of self-renewal and/or differentiation of stem/progenitor cells to drive regeneration (Figure 2e,f) [26,60,62,64,65,66,67,68,69,70,71,72,73,74,75,76]. In the taste bud, the input from the Wnt pathway had a role in the reestablishment of taste cell differentiation upon fractionated IR [75]. IR led to an extensive reduction of progenitor proliferation, an increase in cell death, and a downregulation of the Wnt signaling pathway in mice tongue. Since Wnt activity was recovered during cellular proliferation but before taste cell differentiation, it was suggested that the Wnt pathway is required for taste cellular maturation rather than for proliferation (Figure 2e). Similarly, the Wnt pathway orchestrates SC dependent-mammary tissue repair after IR (Figure 2e) [72]. IR of primary murine mammary epithelial cells (MECs) with or without Wnt1 gain-of-function, enriched the side population and Sca+ stem/progenitor cells. In vivo IR of murine mammary glands induced an increase of Sca+ cells, while decreasing the CD24+CD29+ SC population. Overall, these data suggest that different populations of mammary stem/progenitor cells are mobilized after IR and that the Wnt signaling pathway is involved in radiosensitivity. 

A role of the Wnt pathway in SG during regeneration after IR injury has been suggested. Transplantation of human or mouse submandibular gland organoids derived cells into locally SG IR mice led to cellular engraftment, proliferation, submandibular gland functional regeneration, and recovery of ductal expression of β-catenin and Axin2 [26,77], indicating a role for endogenous paracrine Wnt signals (Figure 2e). In human SGs, the involvement of the Wnt signaling pathway in the damage inflicted on the surviving acinar cells post-IR has been suggested (Figure 2f) [73]. SG tissue atrophy, loss of acinar cells, and upregulation of Wnt1 and β-catenin expression most prominently in the remaining viable acinar cells indicated that Wnt modulation might provide cues for SG remodeling. 

It has been reported that an unbalanced Wnt activity resulting from knockout of genes like Caveolin 1 [60], Focal adhesion kinase [64], c-Myc [64], and PCNA-associated factor [65] might be responsible for an impairment in SC driven intestinal recovery post-IR. Moreover, the role of Wnt signaling under Yap1 modulation in sustaining intestinal repair after IR is discussed in the next section of this review [78,79,80], suggesting important crosstalk of the Wnt pathway with other genes/proteins to orchestrate tissue remodeling. 

In addition to the differences in the cellular turnover and the sensitivity to Wnt modulation, the Lgr5 and Bmi1 populations exhibited distinct responses to IR damage [74]. Abrogation of the proliferation of Lgr5+ ISCs and progeny and expression of the Wnt target gene Olfm4 resulted from 12 Gy whole-body IR of Lgr5 reporter mice, whereas the IR of Bmi1 reporter mice led to the expansion of Bmi1+ ISCs and progeny. The intestine harbors functionally distinct SCs constituting the highly radiosensitive Lgr5+ population and the Bmi1+ ISCs that mostly contribute to repair after IR damage. In contrast with the radiosensitivity of Lgr5 SCs in the small intestine, the opposite seems true for the large intestine [66]. Whole-body IR of 15 and 19 Gy or 19 Gy of targeted IR to the distal colon of Lgr5-lacZ reporter mice resulted in the depletion of small intestine Lgr5+ SCs and crypts whereas degenerating/regenerating crypts remained in the colon. Moreover, Lgr5 SCs from the large intestine repaired DNA damage more efficiently and escaped checkpoint adaptation, resulting in less aberrant mitosis. Therefore, the radioresistance of the murine large intestine was attributed to the capacity of Wnt-driven Lgr5+ SCs to repair DNA damage, leading to the regeneration of the epithelial mucosa. Yamauchi [71] explored the in vitro radiosensitivity of the Wnt-driven Lgr5+ SCs and their progeny after exposure of intestinal organoids to radiation. Moreover, 7.25–4000 mGy IR of single crypt cells from Lgr5 reporter mice resulted in a reduction of the organoid forming efficiency. Increased organoid size and secondary organoid formation were observed after IR with higher doses, indicating enhanced proliferation of surviving SCs. Therefore, the hyperproliferation of Lgr5+ SCs and their progeny were suggested to be responsible for the radiation-induced response. These observations confirmed the in vivo regeneration capacity of surviving Lgr5+ cells to promote repair. Since the Lgr5+ population is normally depleted after in vivo lethal IR of the small intestine, it will be interesting to also analyze the in vitro response of the radioresistant Bmi1+ ISCs, which are multipotent and can also form organoids [74]. These studies describe the importance of Wnt signals in driving the response of different intestinal stem/progenitor populations to achieve regeneration after IR damage (Figure 2e).

Some studies have employed Wnt agonists, factors, or conditional medium containing Wnt ligands, to mitigate radiation-induced damage by promoting tissue regeneration. The prophylactic administration of recombinant Rspo1 reduced IR-induced Lgr5+ small ISC depletion [81], whereas treatment with the anti-neoplastic BCN057, mitigated the intestinal radiation damage through Wnt driven survival and increased Lgr5+ cellular proliferation [68]. However, the potential use of Wnt agonists in the repair of intestinal damage post-IR relied on its timely induction. An additional factor that promoted intestinal regeneration through the modulation of the Wnt pathway is the erythroid differentiation regulator 1 (Erdr1), which is induced by early-life microbiota and expressed by Lgr5+ ISCs and progeny in germ-free (GF) adult mice [69]. Administration of recombinant Erdr1 to irradiated GF mice (but not when colonized with specific-pathogen-free microbiota) or to organoids, prevented the depletion of Lgr5+ cells through Wnt signaling activation. Moreover, the ISC niche damage induced by 11.2 Gy whole-body IR or 18 Gy abdominal IR of mice with a specific deletion of Porcupine (inducer of palmitoylation of Wnt molecules) in macrophages was ameliorated by secreted Wnt5a, 6, and 9a ligands from extracellular vesicles contained in the conditioned medium collected from the culture of macrophages derived from the bone marrow of wild type mice [62]. Therefore, the activation of the Wnt signaling pathway seems like a potential therapeutic approach to promote crypt regeneration of irradiated intestines.

To mimic temporally pharmacological Wnt signaling activation, the in vivo use of shRNA technology has been explored [70]. Indeed, shRNA targeting APC displayed an increase in the number of crypts in 14.5 Gy irradiated small murine intestines, accompanied by upregulation of ISC markers Lgr5 and Ascl2, and Wnt target genes c-Myc and Axin2. Transient Wnt1 activation after head/neck-IR of Krt5-Wnt1 inducible transgenic mice [76] only resulted in increased submandibular gland ductal Sca-1 proliferating cells, whereas concurrent Wnt1 induction improved saliva production, acinar cell survival, and Ascl3 progenitor marker expression. The radioprotection of concurrent Wnt activation was confirmed in vitro, improving the proliferation of the stem/progenitor cells from salispheres derived from irradiated mice. As in the case of the intestine [70], SGSC proliferation to repair tissue could be induced by transient Wnt activation.

In summary, the Wnt pathway plays a crucial role as a driver of tissue remodeling through the initiation of stem/progenitor proliferation. Strategies developed to modulate the Wnt signaling are a potential approach to promote tissue regeneration post-IR.

### 3.4. Hippo Signaling Pathway 

The Hippo signaling pathway is considered a key regulator of organ growth, through the control of cell proliferation and survival [78,82]. The Hippo pathway acts through a kinases cascade composed of the serine/threonine kinases Mst1/2 (mammalian Ste2-like kinases) and Lats1/2 (large tumor suppressor kinase 1/2), which phosphorylate the transcriptional cofactors Yap1 and transcriptional coactivator with Pdz binding motif (Taz), followed by their translocation to the cytoplasm and their degradation in a ubiquitin-proteasome dependent manner. In the absence of Hippo signaling, dephosphorylated Yap1 and Taz translocate into the nucleus and activate the Tead/Tef family of transcription factors [82,83].

The role of the Hippo signaling pathway in the proliferation and function of multiple adult stem/progenitor cells during homeostasis and regeneration has been investigated. Genetic Yap1 activation results in the expansion of stem/progenitor cells and altered differentiation in the homeostatic murine skin [84,85], liver [86,87], lung [88], and the brain [89]. In the developing brain, Yap1 overexpression produced malformation of the murine hippocampus [89]. In the intestine, murine Yap1 activation-induced dysplasia, characterized by Wnt pathway activation and replacement of differentiated enterocytes, goblet, and Paneth cells by multipotent progenitors [84]. Targeted expression of Yap1 in the intestinal epithelium resulted in the loss of proliferating crypts, which led to Wnt signaling pathway inhibition and the loss of Paneth cells [78]. Additionally, specific deletion of Yap1 under the Lgr5 promoter leads to an expansion of the ISC population [78], indicating that the modulation of the Hippo pathway is crucial for the maintenance of the homeostatic stem/progenitor pool in different tissues. 

The nuclear translocation of Yap1 is associated with regeneration after IR damage in many organs. Specifically, the involvement of Yap1 in the stem/progenitor cell response after radiation-induced injury has been described in the intestine [78,79,80], the SGs [90], and the brain [82] (Figure 2g). Knockout of Yap1 led to an increase in radiation-induced apoptosis in crypts and in a Wnt hyperactivity that resulted in the formation of crypts mostly composed of Paneth cells. This phenotype could be rescued in crypt-derived organoids by reducing the Wnt signaling level or by the addition of EGFR ligands [79]. Therefore, Yap1 seems to maintain the ISC pool by inhibiting Wnt signaling and inducing regeneration with the participation of EGF signaling. Similarly, intestinal epithelium-specific Yap1 loss caused crypt hyperproliferation, increased number of Paneth cells, Wnt hyperactivity, and unaltered apoptosis upon IR [78]. This was suggested to be due to Wnt signaling restriction induced by cytoplasmic Yap1, independent of the β-catenin levels promoted by the Axin-APC-GSK-3β complex. Wnt signaling was directly modulated by the Yap1 dependent nuclear localization of Dvl2 in the intestinal stem/progenitor cells. In concordance, it was shown that Yap1 upregulation led to increased intestinal regeneration [80]. Moreover, 10 Gy whole-body IR of mice with a deletion of protein kinase C ζ (Pkcζ) in Lgr5+ SCs promoted upregulation of Yap1 signaling, a higher number of crypts, hyperproliferation of Lgr5+ SCs, and upregulation of nuclear β-catenin through Pkcζ-Axin-APC-GSK-3β induced phosphorylation of Yap1, inhibiting its transcriptional activity.

The activation of Yap1 has been reported to be crucial for progenitor cells during the recovery of the adult cerebellum after IR (Figure 2g) [82]. Knockout of Yap1 in Nestin-expressing progenitors after birth, followed by 4 Gy IR, led to a reduction in cerebellar size, a decrease of the internal granule cell layer, and altered localization of Purkinje cells and Bergmann glial fibers at postnatal day 30. Interestingly, the IR of mice with a knockout of Taz in Nestin progenitors did not alter the size of the cerebellum. The time point of injury and the stage when the response to damage was assessed could have an impact on the resulting observations. Overall, Yang and Joyner [82] highlighted the crucial role of Yap1 in Nestin-expressing progenitors for the orchestration of cerebellar recovery after IR damage during developmental stages.

Interestingly, while Yap1 nuclear translocation is required for regeneration of the intestine and the brain, it seems to hamper tissue repair in the parotid gland (Figure 2g) [90]. A single dose of 5 Gy IR to mice with genetic ablation of Pkcζ reduced the proliferation of label-retaining acinar cells, accompanied by an upregulation of nuclear Yap1 translocation. The functional recovery of saliva production normally achieved by the administration of IGF-1 after IR [91] was absent under Pkcζ ablation. Similarly, 5 Gy of head/neck-IR of FVB mice resulted in higher nuclear Yap1 localization in the acinar compartment while no changes were reported in cells of major ducts [92]. IGF-1 treatment of irradiated mice restored Yap1 nuclear localization to normal, suggesting that the SG functional recovery promoted by IGF-1 is a consequence of the reduction of Yap1 nuclear translocation. However, the use of a localized higher dose of IR [93] might lead to different observations. 

Overall, the presented studies describe the tissue-dependent role of Yap1 nuclear localization as a key regulator in the stem/progenitor cell niche to drive regeneration after IR injury during adulthood.

### 3.5. Autophagy Pathway 

Autophagy is an evolutionarily conserved catabolic pathway that is necessary for maintaining cellular homeostasis by removing damaged proteins and organelles [94,95]. During the induction of the autophagy pathway, sections of the cellular cytoplasm are sequestered into double-membraned structures called autophagosomes. The autophagosome elongation is conducted by the formation of a complex of autophagy protein 5 (Atg5), an E3 ubiquitin ligase, with Atg12 and Atg16L1. Simultaneously, the cytosolic microtubule-associated protein 1A/1B-light chain 3 (Lc3/Atg8)-I is conjugated to phosphatidylethanolamine to form Lc3-II, which is recruited to the autophagosomal membranes. The autophagosome formation is followed by its fusion with lysosomes to drive the degradation of their content by lysosomal enzymes [95]. 

The autophagy pathway has been shown as crucial for the maintenance of stem/progenitor cell homeostasis in tissues, such as the intestine [95,96,97] and the SGs [98]. In the intestinal epithelium, specific deletion of Atg5 in Lgr5 ISCs led to an increase of intracellular ROS resulting in fewer ISCs and transient amplifying (TA) cells [95]. Atg7 ISC cKO promoted the induction of apoptosis driven by DNA damage of Lgr5+ ISCs and the reduction of organoid forming efficiency of the crypts [96]. Similarly, the survival of intestinal crypt organoids was reduced by the deletion of ATG16L1 in ISCs [97]. In SG, the acinar-Aqp5 deficiency of Atg5 resulted in acinar hypertrophy with an accumulation of secretory granules, which led to further acinar enlargement after administration of isoproterenol [98]. These and other data showed that autophagy is necessary for the control of homeostasis driven by some tissue-specific SCs.

Some studies have described a crucial involvement of autophagy in radiation-induced regeneration of SGs [94,99], intestine [95,100], and kidney [101] (Figure 2h). Morgan-Bathke et al. [94] explored the role of autophagy in parotid gland IR-triggered repair of acinar cells (Figure 2h), a population that is known to self-duplicate to contribute to endogenous regeneration [22]. At first, targeted 5 Gy head/neck-IR of mice with a cKO of Atg5 in Aqp5 expressing acinar cells (autophagy-deficient mice) led to a persistent reduction in saliva flow, decreased amylase production, and increased apoptosis. IR of wild type mice did not induce autophagy unless IGF-1 was administered while IGF-1 treatment of autophagy-deficient mice failed to restore their SG function. Since IGF-1 administration prevented the sequestration of Ambra1 by Bcl-2, activating autophagy and inhibiting apoptosis, it was postulated that autophagy is necessary for parotid gland regeneration. Based on these observations, the author further explored the potential induction of autophagy to reduce SG radiation damage [99]. To this end, the rapamycin analog CCI-779 was used as an autophagy activator, which inhibits the mTOR complex 1 (mTORC1), followed by the sequestration of Atg13. CCI-779 post-IR treatment of 5 Gy head/neck irradiated wild type mice improved parotid gland tissue integrity, increased saliva production, preserved the expression of amylase, and normalized cellular proliferation. The last being crucial for the maintenance of the poll of acinar cells, since autophagy-deficient mice showed an elevated proliferation response that correlated with poor SG function. On the other hand, the use of the autophagy inhibitor chloroquine did not rescue the saliva production. Similarly, the administration of Rapamycin induced renal protection against radiation damage (Figure 2h) [101]. Kidneys of mice exposed to 8 Gy of total body IR showed a reduced expression of the renal SC marker CD133, activation of mTORC1 signaling, and inhibition of autophagy, while rapamycin treatment ameliorated renal morphological damage, increased CD133 expression, reduced apoptosis, and inhibited the overactivation of TGF-β and NF-κB. These data suggest that the activation of autophagy rescued kidney tissue integrity by promoting the density of SCs that could support regeneration post-IR. Furthermore, Levy described in vivo and in vitro beneficial effects of using an initiator of autophagy as a radioprotector of ISCs (Figure 2h) [100]. The employed agent was the bacterial peptidoglycan motif nucleotide-binding oligomerization domain-containing protein 2 (NOD2) agonist muramyl dipeptide (MDP), which recruits Atg16L1 to the plasma membrane and promotes the induction of autophagosome formation. Pre-treatment with MDP of 2 Gy-irradiated murine small intestinal mature organoids led to an increase of their survival, a higher number of surviving LGR5+ ISCs, a decrease of cleaved caspase-3+ ISCs, reduction of mitochondria, and preservation of ROS levels. This protection was not observed in organoids derived from NOD2 and Atg16L1 deficient mice, however, it was partially restored by the antioxidant resveratrol. In vivo MDP treatment pre-total body IR of Lc3 reporter mice depleted from intestinal microbiota caused the induction of autophagy and mitophagy. Therefore, the observed intestinal regeneration after IR was attributed to the MDP-NOD2 mediated Atg16L1 activation of mitophagy and the control of intracellular ROS levels in ISCs. Asano and colleagues [95] further supported the protection against ROS intestinal cytotoxicity through mitophagy stimulation. Specifically, 10 Gy whole-body IR of mice harboring a deletion of Atg5 in the Lgr5+ ISCs led to few viable crypts, the formation of blunt villi, and reduction of organoid forming efficiency of the crypts. This ISC radiosensitization also has been reported after Atg7 deletion in the intestinal epithelium, which increased apoptotic DNA damage in Lgr5+ cells after 10 Gy IR [96]. The drastically impaired intestinal regeneration in irradiated Atg5 deficient mice was restored by the treatment with the antioxidant N-acetyl-L-cysteine, increasing the number of proliferative ISCs and TA cells [95]. As in the study of Levy [100], it was considered that autophagy plays an important role in driving intestinal regeneration by balancing the intracellular ROS levels in the SC population.

To conclude, these studies describe that the loss of autophagy impairs the regeneration of different adult tissues after IR damage and that the activation of autophagy seems to be a beneficial approach to induce the SC repair response to radiation.

## 4. Conclusions

RT constitutes the primary line of treatment for many different cancers. Unfortunately, the normal tissue may be damaged by the co-IR resulting in functional impairment of the affected organ. Evidence suggests that homeostasis and regeneration of many adult tissues are supported by residing stem/progenitor cells which are targeted by the IR treatment, hampering their capacity to promote tissue repair and functionality. The niche of these stem/progenitor cells is tightly regulated by the input of different signaling pathways however, the regulatory mechanisms that drive the SC/progenitor response during IR damage are still not completely understood. We discussed the Notch, Hh, Wnt, Hippo, and autophagy as the most important signaling cascades that are crucial for driving sustained stem/progenitor regeneration after RT in different solid adult tissues (Figure 2). Concisely, the modulation of these signaling pathways offers a potential tool for the preservation and stimulation of the activity of stem/progenitor cells after RT. However, different aspects must be considered before translating this therapeutic approach towards the clinic. At first, the desired targeted stem/progenitor population has to be determined since there is evidence of specific stem/progenitor activity after the modulation of some of these signaling pathways. Additionally, reported differences regarding the irradiated area and the time frame of induction imply a careful interpretation of the in vivo derived results and a crucial selection of the timing of the treatment. Importantly, the modulation of SC signaling pathways to achieve normal tissue protection should not induce a radioresistance phenotype in cancer cells. Indeed, a critical role of the Notch, Hh, Wnt, and autophagy pathway in cancer progression has been indicated. Therefore, these signaling pathways offer potential genes/protein as therapeutic targets, which modulation must be carefully spatiotemporal regulated to achieve stem/progenitor driven regeneration after RT, without compromising the antitumoral effects of RT.

We described data clearly showing species differences regarding the stem/progenitor response to IR. This situation highlights the need for further research using human tissue to revalidate the murine data. For this, novel models are needed. We described the current models employed to study the stem/progenitor response to IR. The organoid technology and the ALI system seem to be the most suitable in vitro tools, attributable to their tissue structure and functionality resemblance, versatility, and human tissue derivation. However, the influence of the organ complexity can be only addressed through the use of in vivo preclinical models, which have the principal disadvantage of needing extrapolation to the human situation. Therefore, advances in the complexity of the current models should be considered to obtain data that more accurately describe the mechanisms that govern normal tissue response to IR. To conclude, this review discussed currently available models used for the study of the stem/progenitor cellular response to RT and the crucial signaling pathways involved during adult tissue radiation-induced regeneration orchestrated by solid tissue-resident stem/progenitor cells.
